# DNA barcoding as a screening tool for cryptic diversity: an example from *Caryocolum*, with description of a new species (Lepidoptera, Gelechiidae)

**DOI:** 10.3897/zookeys.404.7234

**Published:** 2014-04-24

**Authors:** Peter Huemer, Ole Karsholt, Marko Mutanen

**Affiliations:** 1 Tiroler Landesmuseen Betriebgsges.m.b.H., Naturwissenschaftliche Sammlungen, Feldstr. 11a, A-6020 Innsbruck, Austria; 2 Zoological Museum, Natural History Museum of Denmark, Universitetsparken 15, DK-2100 Copenhagen; 3 Biodiversity Unit, Department of Biology, University of Oulu, Oulu, Finland

**Keywords:** Lepidoptera, Gelechiidae, *Caryocolum*, species delineation, integrative taxonomy, DNA barcode, morphology, Europe

## Abstract

We explore the potential value of DNA barcode divergence for species delimitation in the genus *Caryocolum* Gregor & Povolný, 1954 (Lepidoptera, Gelechiidae), based on data from 44 European species (including 4 subspecies). Low intraspecific divergence of the DNA barcodes of the mtCOI (*cytochrome c oxidase 1*) gene and/or distinct barcode gaps to the nearest neighbor support species status for all examined nominal taxa. However, in 8 taxa we observed deep splits with a maximum intraspecific barcode divergence beyond a threshold of 3%, thus indicating possible cryptic diversity. The taxonomy of these taxa has to be re-assessed in the future. We investigated one such deep split in *Caryocolum amaurella* (Hering, 1924) and found it in congruence with yet unrecognized diagnostic morphological characters and specific host-plants. The integrative species delineation leads to the description of *Caryocolum crypticum* **sp. n.** from northern Italy, Switzerland and Greece. The new species and the hitherto intermixed closest relative *C. amaurella* are described in detail and adults and genitalia of both species are illustrated and a lectotype of *C. amaurella* is designated; a diagnostic comparison of the closely related *C. iranicum* Huemer, 1989, is added.

## Introduction

The genus *Caryocolum* Gregor & Povolný, 1954 is one of the most species-rich genera of European Gelechiidae (Huemer and Karsholt 2010). Having been revised in monographic papers (Klimesch 1953–1954, Huemer 1988), its taxonomy seemed well established. However, in the last decade new species were found in, e.g. Sicily, southern France and Greece (Bella 2008, Grange and Nel 2012, Huemer and Nel 2005, Huemer and Karsholt 2010) raising the number of described species to 51. Most of the species are considered indisputable based on their morphology and distinct biology – as far as known, these species are closely linked to Caryophyllaceae as their exclusive larval host-plant family. We investigate, for the first time in *Caryocolum*, the congruence of traditional morphological species delineation and molecular data from the COI barcode region for a vast majority of the European fauna, covering altogether 44 species, including four subspecies. Surprisingly, the potential for cryptic diversity proved extraordinarily high for a supposedly well-known genus and we newly describe one of the hitherto overlooked species.

## Material and methods

Extensive generic descriptions and diagnoses of European species of *Caryocolum* have been published in several reviews, particularly Huemer and Karsholt (2010) and Huemer (1988), and are thus not repeated here.

Specimens. Our study is based on about 50 specimens of the *Caryocolum amaurella* (Hering, 1924) species-group and an uncounted number of European *Caryocolum*, exceeding 1000 specimens, but only partially used for genetic analysis (see below). Most of the material was traditionally set and dried or alternatively spread; a few specimens are only pinned. Genitalia preparations followed standard techniques (Robinson 1976) adapted for male genitalia of Gelechiidae and (some) female genitalia of *Caryocolum* by the so-called “unrolling technique” (Pitkin 1986, Huemer 1987).

DNA Barcodes. Full-length lepidopteran DNA barcode sequences are a 648 base-pair long segment of the 5’ terminus of the mitochondrial COI gene (*cytochrome c oxidase 1*). DNA samples (dried leg) were prepared according to the accepted standards. Legs from 250 specimens of *Caryocolum* were processed at the Canadian Centre for DNA Barcoding (CCDB, Biodiversity Institute of Ontario, University of Guelph) to obtain DNA barcodes using the standard high-throughput protocol described in deWaard et al. (2008). Sequences longer than 500 bp were included in the analysis. Successfully sequenced voucher specimens are listed in Suppl. material 1. Sequences were submitted to GenBank; further details including complete voucher data and images can be accessed in the public dataset “Lepidoptera of Europe *Caryocolum*” dx.doi.org/10.5883/DS-LECARY in the Barcode of Life Data Systems (BOLD; Ratnasingham and Hebert 2007). Degrees of intra- and interspecific variation in the DNA barcode fragment were calculated under Kimura 2 parameter (K2P) model of nucleotide substitution using analytical tools in BOLD systems v3.0. (http://www.boldsystems.org). A neighbour-joining tree of DNA barcode data of European taxa was constructed using Mega 5 (Tamura et al. 2011) under the K2P model for nucleotide substitutions.

Photographic documentation. Photographs of the adults were taken with an Olympus SZX 10 binocular microscope and an Olympus E 3 digital camera and processed using the software Helicon Focus 4.3 and Adobe Photoshop CS4 and Lightroom 2.3. Genitalia photographs were taken with an Olympus E1 Digital Camera from Olympus BH2 microscope.

### Abbreviations of institutional collections

BMNH The Natural History Museum (British Museum, Natural History) London (United Kingdom)

TLMF Tiroler Landesmuseum Ferdinandeum, Innsbruck, Austria

ZMUH Zoological Museum, University of Helsinki, Finland

ZMUC Zoological Museum, Natural History Museum of Denmark, Copenhagen, Denmark

ZMUO Zoological Museum, University of Oulu, Finland

## Results

### Molecular analysis

**Figure 1.**
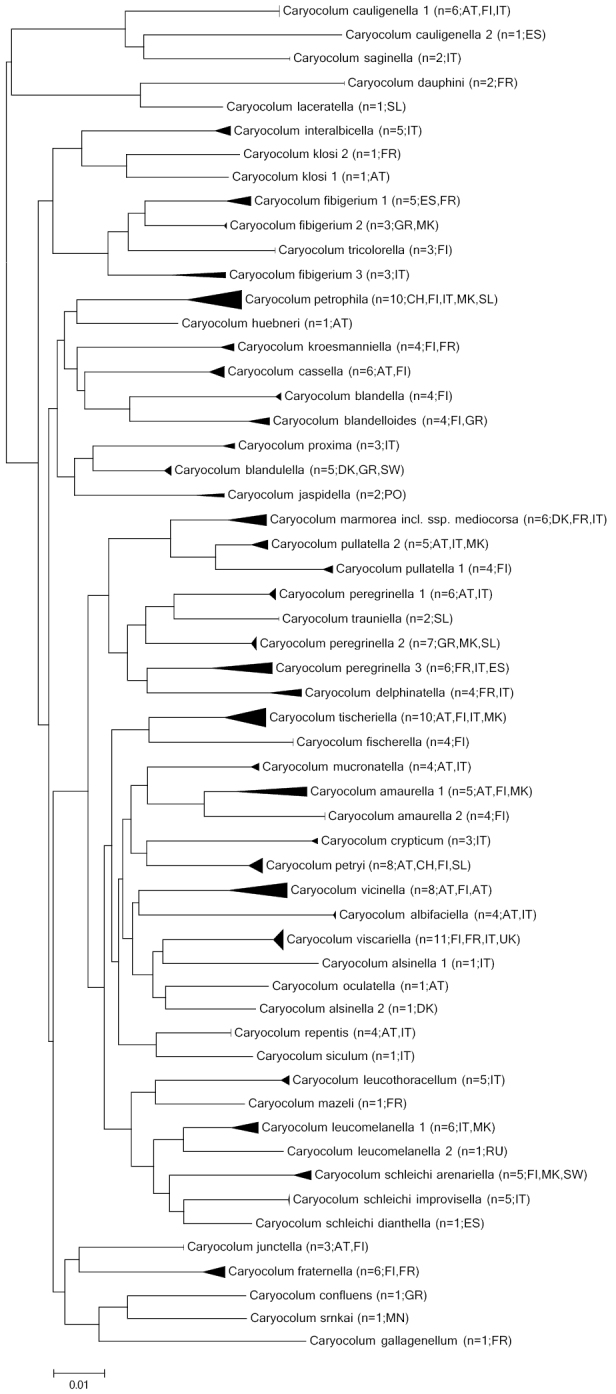
Neighbour-joining tree (Kimura 2 parameter, built with MEGA 5; cf. Tamura et al. 2011), with only sequences longer than 500 bp considered. The width of the triangles represents the sample size, and the depth the genetic variation within the cluster. Currently recognized conspecific taxa with maximum divergence greater than 3% are shown as separate clades. Source: DNA Barcode data from BOLD (Barcode of Life Database, cf. Ratnasingham and Hebert 2007).

Forty-four of 51 European species were successfully sequenced, resulting in a full-length barcode fragment for 191 specimens and more than 500 bp for further 26 specimens (Fig. 1, Table 1, Suppl. material 1). Nine shorter sequences were not included in the analysis and sequencing of 24 specimens failed. The maximum intraspecific K2P distance varies from 0% in several species to 6.27% in *Caryocolum fibigerium*. Ten species have a high maximum intraspecific divergence greater than 2%. In six species (newly described species excluded) with a medium divergence greater than 3% potential cryptic diversity should be investigated. Furthermore, the intraspecific divergence of more than 3% in *Caryocolum schleichi*, a species separated into 3 allopatric subspecies, is beyond variation typically found within species, supporting their status as valid species. The only other subspecies we have examined are nominotypical *Caryocolum marmorea* and the recently separated *Caryocolum marmorea mediocorsa* with a very low divergence of 0.3%.

**Table 1.** Intraspecific mean K2P (Kimura 2 Parameter) divergences, maximum pairwise distances and distance to nearest neighbor.

Species	Mean Intra-Sp	Max Intra-Sp	Nearest Neighbour	Nearest Species	Distance to NN
*Caryocolum alsinella*	4.85	4.85	PHLAE427-11	*Caryocolum oculatella*	3.81
*Caryocolum amaurella*	3.05	4.76	LEATC402-13	*Caryocolum mucronatella*	5.21
*Caryocolum blandella*	0.16	0.3	LEFIK150-10	*Caryocolum blandelloides*	5.78
*Caryocolum blandelloides*	0.4	0.81	LEFIB755-10	*Caryocolum blandella*	5.78
*Caryocolum blandulella*	0.21	0.46	LEATD656-13	*Caryocolum proxima*	3.94
*Caryocolum cassella*	0.42	0.61	PHLAI019-12	*Caryocolum blandulella*	5.07
*Caryocolum cauligenella*	1.99	6.95	PHLAA069-09	*Caryocolum saginella*	6.61
*Caryocolum confluens*	N/A	N/A	PHLAF489-11	*Caryocolum srnkai*	4.54
*Caryocolum crypticum*	0.21	0.31	LEATC-402-13	*Caryocolum mucronatella*	5.41
*Caryocolum dauphini*	0	0	PHLAB900-10	*Caryocolum laceratella*	5.29
*Caryocolum delphinatella*	1.02	1.39	PHLAI203-13	*Caryocolum marmorea mediocorsa*	4.57
*Caryocolum fibigerium*	3.4	6.27	LEFIF467-10	*Caryocolum tricolorella*	4.67
*Caryocolum fischerella*	0	0	LEFIC281-10	*Caryocolum tischeriella*	4.5
*Caryocolum fraternella*	0.47	1.7	PHLAI156-12	*Caryocolum junctella*	4.55
*Caryocolum gallagenellum*	N/A	N/A	PHLAI019-12	*Caryocolum blandulella*	6.54
*Caryocolum huebneri*	N/A	N/A	LEFIJ1014-11	*Caryocolum petrophila*	4.88
*Caryocolum interalbicella*	0.4	0.77	PHLAI156-12	*Caryocolum junctella*	5.55
*Caryocolum jaspidella*	1.08	1.08	PHLAI019-12	*Caryocolum blandulella*	4.39
*Caryocolum junctella*	0	0	LEFIF480-10	*Caryocolum fraternella*	4.55
*Caryocolum klosi*	4.25	4.25	PHLAA055-09	*Caryocolum interalbicella*	5.56
*Caryocolum kroesmanniella*	0.31	0.61	LEEUA184-11	*Caryocolum blandulella*	4.9
*Caryocolum laceratella*	N/A	N/A	PHLAI447-13	*Caryocolum dauphini*	5.29
*Caryocolum leucomelanella*	1.47	3.79	PHLAG331-12	*Caryocolum mazeli*	3.76
*Caryocolum leucothoracellum*	0.12	0.3	PHLAG331-12	*Caryocolum mazeli*	4.24
*Caryocolum marmorea mediocorsa*	0	0	LEEUA182-11	*Caryocolum marmorea*	0.3
*Caryocolum marmorea*	1	1.54	PHLAI203-13	*Caryocolum marmorea mediocorsa*	0.3
*Caryocolum mazeli*	N/A	N/A	LEATE421-13	*Caryocolum leucomelanella*	3.76
*Caryocolum mucronatella*	0.3	0.46	PHLAE427-11	*Caryocolum oculatella*	4.87
*Caryocolum oculatella*	N/A	N/A	LEEUA388-11	*Caryocolum alsinella*	3.81
*Caryocolum peregrinella*	3.58	5.69	PHLAB899-10	*Caryocolum trauniella*	3.93
*Caryocolum petrophila*	0.97	2.26	PHLAH147-12	*Caryocolum huebneri*	4.88
*Caryocolum petryi*	0.23	0.61	PHLAD576-11	*Caryocolum repentis*	3.85
*Caryocolum proxima*	0.41	0.61	PHLAI019-12	*Caryocolum blandulella*	3.94
*Caryocolum pullatella*	2.07	3.61	LEATC292-13	*Caryocolum marmorea*	3.12
*Caryocolum repentis*	0	0	PHLAE429-11	*Caryocolum siculum*	3.33
*Caryocolum saginella*	0	0	LEFIJ778-10	*Caryocolum cauligenella*	6.61
*Caryocolum schleichi dianthella*	N/A	N/A	PHLAD573-11	*Caryocolum schleichi improvisella*	3.42
*Caryocolum schleichi improvisella*	0.06	0.15	PHLSA085-11	*Caryocolum schleichi dianthella*	3.42
*Caryocolum schleichi arenariella*	0.77	1.24	PHLSA085-11	*Caryocolum schleichi dianthella*	3.74
*Caryocolum siculum*	N/A	N/A	PHLAD576-11	*Caryocolum repentis*	3.33
*Caryocolum srnkai*	N/A	N/A	PHLAG580-12	*Caryocolum confluens*	4.54
*Caryocolum tischeriella*	1.09	2.02	PHLAD576-11	*Caryocolum repentis*	4.01
*Caryocolum trauniella*	0	0	PHLAB622-10	*Caryocolum peregrinella*	3.93
*Caryocolum tricolorella*	0	0	PHLAI014-12	*Caryocolum fibigerium*	4.67
*Caryocolum vicinella*	1.48	2.7	PHLAF105-11	*Caryocolum leucomelanella*	5.36
*Caryocolum viscariella*	0.22	0.47	LEEUA388-11	*Caryocolum alsinella*	4.16

Sequences of the COI barcode region of all analysed morphospecies reveal significant interspecific genetic distances with barcode gaps ranging from a minimum of 3.11% to the nearest neighbour (*Caryocolum pullatella* – *Caryocolum marmorea*) to a maximum of 6.61% (*Caryocolum saginella* – *Caryocolum cauligenella*).

## Taxonomy

The *Caryocolum amaurella* species-group as defined by Huemer (1988) differs from other congeners mainly by the characteristic shape of the sacculus, which is unique in the genus. Until now it only included *Caryocolum amaurella* and *Caryocolum iranicum* (Huemer 1988, 1989b). Based on the DNA barcode divergence and diagnostic morphological characters combined with biological data we describe the new species *Caryocolum crypticum*. Due to the mix-up of *Caryocolum crypticum* with *Caryocolum amaurella* in recent identification guides the latter species is also re-described here in detail.

### *Caryocolum* Gregor & Povolný, 1954

*Caryocolum* Gregor & Povolný, 1954: 87.

**Type species.** *Gelechia leucomelanella* Zeller, 1839: 138.

#### Caryocolum crypticum sp. n.

http://zoobank.org/5E1FB9E5-3A65-49C6-80BF-A5CA7C4FFF99

http://species-id.net/wiki/Caryocolum_crypticum

Figs 2–3 , 6–7 , 10–11 , 14–15

##### Type material.

**Figures 2–5.**
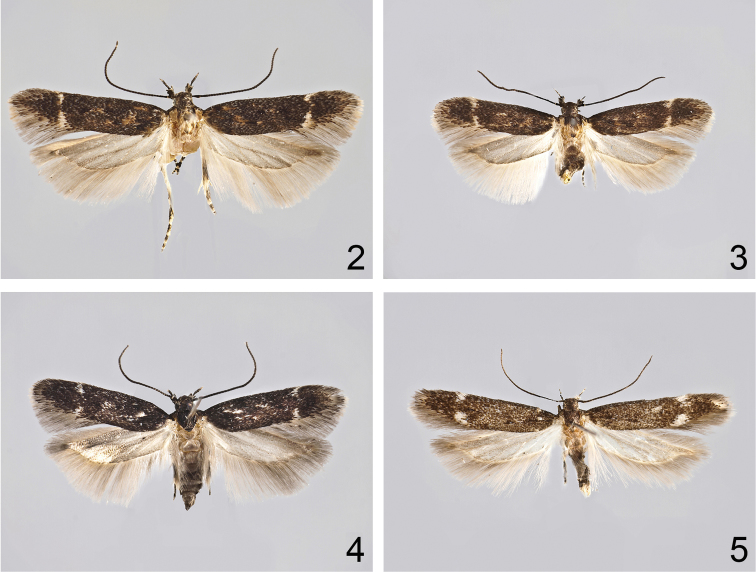
Adults. **2** *Caryocolum crypticum* sp. n., holotype **3** *Caryocolum crypticum* sp. n., paratype, female, Greece **4** *Caryocolum amaurella*, male, Finland **5** *Caryocolum amaurella*, male, Austria.

**Holotype:** ♀ (Fig. 2), Italia sept., Teriolis merid., Laatsch, 1000 m, 29.6.1987 e.l. (*Silene otites* 10.5.), leg. Huemer, slide GEL 1234 ♀ (TLMF).

**Paratypes.** **Italy:** 1 ♂, South Tyrol, Vinschgau, Schleiser Leiten, 1350 m, 6.7.2013, leg. Huemer, slide GEL 1215, dna barcode id TLMF Lep 12313 (TLMF); 1 ♂ [without abdomen], same data (TLMF); 1 ♀, same data, but 18.8.2013, slide GEL 1232, dna barcode id TLMF Lep 11883 (TLMF); 1 ♀, same data, but dna barcode id TLMF Lep 11882 (TLMF); 1 male [without abdomen], 8 ♀, same data, but 7.9.2013 (TLMF); 1 ♂, South Tyrol, Taufers, 1300 m, 22.8.1978, leg. Burmann, slide GU 86/041 P. Huemer (TLMF). **Switzerland:** 2 ♀, Wallis, Martigny-Rosel, 460 m, 28.6.–14.7.1983 e.l. (*Silene otites*), leg. Whitebread (Naturhistorisches Museum Basel, Switzerland). **Greece:** 1 ♀, Larisa, Ossa Oros, 1.5 km N Spilia, 940 m, 13.6.1988 e.l. (*Silene nutans*), leg. Huemer (TLMF).

##### Diagnosis.

**Figures 6–7.**
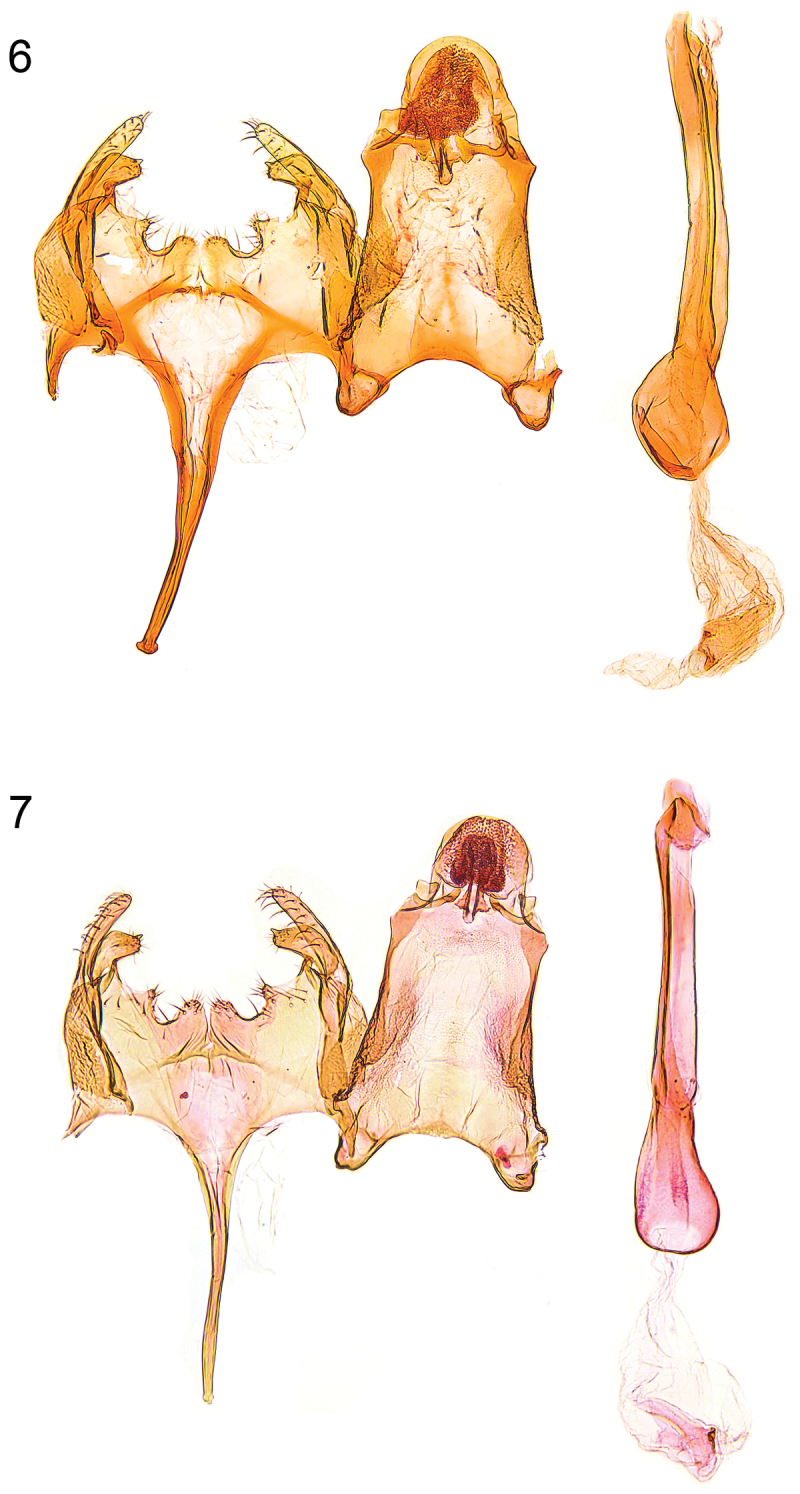
Male genitalia. **6** *Caryocolum crypticum* sp. n., paratype, Italy, slide GU 86/041 P.Huemer **7** *Caryocolum crypticum* sp. n., paratype, Italy, slide GEL 1215 P.Huemer.

*Caryocolum crypticum* sp. n. is externally similar to several other species of the genus and can be best recognized by the largely unmarked forewings with cream costal and tornal spots. From its closest relatives *Caryocolum amaurella* and *Caryocolum iranicum* it differs by the rusty brown distal half of the thorax and the concolorous tegulae, the dark brown forewings with rusty brown scales, and the cream colours of the costal and tornal spots. The male genitalia of *Caryocolum crypticum* are very similar to those of *Caryocolum amaurella* but the valva is more slender and slightly longer (see Figs 6–7, 10–11 versus 8–9, 12–13). The similar *Caryocolum iranicum* differs by the shape of the sacculus with almost straight dorsal margin (see Huemer 1989b: Figs 14–16). However, the most striking diagnostic characters of the new species are found in the female genitalia which differ from *Caryocolum amaurella* particularly by the short lateral sclerites of the ductus bursae and the much longer and more slender signum hook (see Figs 14–15 versus 16–17). The female genitalia furthermore differ from *Caryocolum iranicum* by the weakly cup-shaped rather than funnel-shaped antrum, shorter lateral sclerites of the ductus bursae, and the shorter apophysis anterior which is almost twice the length of segment VIII in *Caryocolum iranicum*.

##### Description.

Adult (Figs 2–3). Wingspan 10.5-14 mm. Segment 2 of labial palpus with a few cream-coloured scales on inner and upper surface, blackish brown on outer and lower surface; segment 3 almost black with light tip. Antenna black, indistinctly lighter ringed. Head with light yellow frons and black neck; thorax blackish brown with rusty brown posterior part; tegulae rusty brown except for blackish brown base. Forewing blackish brown, mottled with some rusty brown, particularly in proximal half; supplementary black spots in fold and in cell obscure; costal and tornal spot small, cream, separated. Hindwing light grey.

Variation. No variation observed except for size, which differs considerably in two reared specimens from Italy and Greece.

**Male genitalia** (Figs 6–7, 10–11). Uncus subovate; tegumen stout; transtilla membranous; valva moderately short and slender, digitate, apex rounded; sacculus short, with angular ventral and weakly convex dorsal margin, apically pointed; posterior margin of vinculum with deep medial emargination and slight medial incision, two pairs of short processes developed; saccus long, comparatively broad at base, distal part gradually tapered; phallus long and slender, weakly curved, with some minute cornuti apically.

**Female genitalia** (Figs 14–15). Segment VIII without processes, subgenital plate sub-triangular, with numerous narrow folds, separated from sclerotized lateral plates by membranous zone; apophysis anterior about length of segment VIII; antrum short, about one quarter length of apophysis anterior, nearly cup-shaped; posterior part of ductus bursae with pair of short sclerites, extending to middle of apophysis anterior, and with two tiny sclerites anteriorly; signum with crescent-shaped base, long and slender, strongly bent hook.

**Molecular data.** The intraspecific divergence of the barcode region is low with mean intraspecific divergence of 0.21% and maximum intraspecific divergence of 0.31% (n=3). The distance to the nearest neighbour *Caryocolum mucronatella* is 5.41%, the divergence to the morphologically closest *Caryocolum amaurella* is 6.82%.

##### Etymology.

The name “*crypticum*” refers to the cryptic morphology of the species and is derived from the latinized adjective *crypticus*.

##### Distribution.

The species is known from widely separated localities in northern Italy, Switzerland and Greece, indicating a more widespread distribution in Sub-Mediterranean and Mediterranean Europe. However, the host-plants are much more widespread, ranging to northern Europe in the north and to Central Asia in the east. No sympatric occurrence with *Caryocolum amaurella* is reported though the two taxa can occur close to one another in the Alps.

##### Bionomics.

The larva has been found in early spring, feeding in the stem of *Silene otites* (L.) Wibel (Caryophyllaceae) (Burmann 1990) and *Silene nutans* L. (Huemer 1989) but detailed descriptions of feeding habits and larval morphology are missing. The adult occurs from early July (reared material dates from mid-June to mid-July) to September and it is attracted to light. *Caryocolum crypticum* prefers xerophilous steppes and rocky habitats with sparse vegetation. Vertical distribution: from about 500 to 1300 m, restricted to mountainous areas.

##### Remarks.

Huemer (1988) already examined females reared from *Silene otites* in Switzerland by Whitebread but in the absence of males considered them as deviating *Caryocolum amaurella*.

The majority of collected material belongs to females whereas *Caryocolum amaurella* is mainly known from the male sex. This may indicate differences in attraction to artificial lights or a female-biased sex ratio in *Caryocolum crypticum*.

#### Caryocolum amaurella

(Hering, 1924)

http://species-id.net/wiki/Caryocolum_amaurella

Figs 4–5 , 8–9 , 12–13 , 16–17

Lita amaurella Hering 1924: 82, Figs 11–12.
Lita viscariae Schütze 1926: 171.

##### Material examined.

Lectotype ♂[with nine labels]: ‘Fennia Ab Bromarf’ ‘R. F:tius’ ‘21.7.21’ [piece of celluloid where genitalia was mounted] ‘Type ♂’ [red] ‘Lita amaurella m. det. Mart. Hering ♂’ ‘Mus. Zool. H:fors spec. typ. No 7016 Lita amaurella Hering’ ‘Lita amaurella m. ♂ Sch.-Armatur Bromarf 21.7.21 Fabritius’ ‘LECTOTYPE O. Karsholt design.’.

**Finland:** 1 ♂, Ab, Naantali, 25.8.1965, leg. Karvonen, slide Karsholt 2719; 2 ♂, N, Ekenäs, 17.–20.7.1980, leg. Fibiger; 1 ♂, N, Helsinki, 25.7.1982, leg. Schnack; 1 ♂, N, Borgå lk., Tirmo, 19–20.7.1980, leg. Fibiger; 5 ♂, same data, but 1.–2.8.1982, leg. Schnack; 2 ♂, U, Sluntle, 18.–31.7.1982, leg. Karsholt (all ZMUC); 6 ♂, 5 ♀, U, Porvoo, 6698:3426 Ånäs, e.l. 2012 (*Lychnis viscaria*), leg. Hirvonen (ZMUO); 4 ♂, V, Dragsfjärd, 664:3249, 2008, leg. Mutanen & Välimäki (ZMUO); 1 ♂, U, Hanko, 6642:3289, 2007, leg. Mutanen & Välimäki (ZMUO). **Sweden:** 2 ♂, Sk, Maglehen, 10.7.1965, leg. Svensson (TLMF, ZMUC); 1 ♂, Sm, Högsby, 13.7.1968, leg. Johansson; 1 ♂, Öl, Ödeshög, 17.7.1972, leg. Karsholt, slide Karsholt 1806; 1 ♂, St. Alvar, Tornrör, 25.7.1997, leg. Hendriksen, slide Hendriksen 1953; 2 ♂, Öl, Gårdby, 2.8.1999, leg. Hendriksen, slide Hendriksen 2411, 2415; 1 ♂, same data, but 22.7.2000; 1 ♂, Gtl., Hejnum Häller, 30.7.1977, leg. Hendriksen, slide Hendriksen 1944; 1 ♂, Ög, Ödeshög, 17.7.1972, leg. Karsholt; Upl., Film, 12.7.1995, leg. Hendriksen (all ZMUC). **Norway:** 2 ♂, On, Vinstra, 19.–29.7.1983, leg. Karsholt & Michelsen, slide Karsholt 4294, 4295; 2 ♀, same data, but 4.–5.7.1987, leg. Karsholt, slide Hendriksen 2099; 2 ♂, same data, but 9.8.1996, leg. Hendriksen (all ZMUC). **Denmark:** 1 ♂, Bornholm, Rø, 7.1892, leg. Gudmann, slide Wolff 2593; 5 ♂, 1 ♀, same data, but 28.7.1978, leg. Schnack, slide Schnack 1118; 1 ♂, Bornholm, Gudhjem, 1 ♂, 2 ♀, 29.6–3.7.1920, leg. Gudmann, slide Wolff 2625, 3682; 1 ♂, 1 ♀, same data, but e.l. 5.1921 (*Lychnis viscaria*), bred 21. & 28.6.1921, leg. Gudmann, slide Wolff 3681(all ZMUC); 6 ♂, 8 ♀, Bornholm, Hammeren, 18.7.1977, leg. Karsholt & Schnack, slide Hendriksen 1767, Karsholt 2948 TLMF, ZMUC); 2 ♂, same data, but 25.7.1977, leg. Schnack; 4 ♂, same data, but 16. –25.7.1978, leg. Schnack; 4 ♂, same data, but 19.–22.7.1979, leg. Hendriksen; 6 ♂, same data, but 29 –30.7.1981, leg. Hendriksen, slide Hendriksen 385, 561, 722; Bornholm, Randkløve, 1 ♂, 22.7.1977, leg. Schnack; Bornholm, 1 ♀, Ringe Bakker, 16.7.1978, leg. Schnack (all ZMUC). **Germany:** 1 ♂, 1 ♀, Lausitz, Umg. Bautzen, e.l. 1935 (*Lychnis viscaria*), leg. Starke (BMNH); 2 ♂, Thüringen, Bad Blankenburg, 14.7.1964, leg. Steuer (TLMF); 1 ♂, Thüringen, Bad Blankenburg, 8.7.1972, leg. Steuer (TLMF). **France:** 2 ♂, Alpes Maritimes, Col de la Cayolle, 2200–2300 m, 29.–30.7.2005, leg. Skou, slide Hendriksen 5364 (ZMUC). **Austria:** 1 ♂, Niederösterreich, Jauerling, 24.7.1935 (TLMF); 2 ♂, Oberösterreich, Windischgarsten, Veichltal, 23.7.1976, leg. Wimmer (TLMF); 1 ♂, Oberösterreich, Waldhausen, Schwarzenberg, 6.8.1997, leg. Wimmer (TLMF); 9 ♂, Kärnten, St. Jakob im Lesachtal, Mussen E, 1680–1800 m, 4.8.1999, leg. Huemer & Erlebach (TLMF). **Slovakia:** 1 ♀, Pol’ana, 28.7.1989, leg. Patocka (ZMUC). **Macedonia:** 4 ♂, NP Mavrovo, Korab, Korabska jezero, Kobilino pole, 2080–2180 m, 28.7.–1.8.2011, leg. Huemer & Tarmann (TLMF). **Turkey:** 2 ♂, 1 ♀, prov. Sivas, 10 km W Görün, 1650 m, 27.7.1989, leg. Esser & Fibiger, slide Huemer GU 90/130, GU 91/215; 4 ♂, prov. Erzerum, Kop Pass, 1750 m, 15.–16.9.1993, leg. Fibiger, slide Hendriksen 2889, 2894; 1 ♂, prov. Erzincan, Kizildaĝ, Geçidi, 2100 m, 19.8.1993, leg. Schepler, slide Hendriksen 2384 (all ZMUC).

##### Diagnosis.

See above.

##### Description.

Adult (Figs 4–5). Wingspan 10–14 mm. Segment 2 of labial palpus bone-white on inner and upper surface, blackish grey on outer and lower surface; segment 3 almost black with light tip. Antenna black, indistinctly lighter ringed. Head with light yellow frons and black neck; thorax and tegula black mottled with brown. Forewing blackish grey mottled with some light brown; base black; two indistinct black spots in fold; one oblique spot above it and one in cell; some white scales before and after these spots; costal and tornal spot small, white, rarely fused. Hindwing light grey.

Variation. The colour of the forewings varies from greyish to blackish. Worn specimens look lighter than fresh ones. Sometimes there are no white scales in the middle of the wing.

**Figures 8–9.**
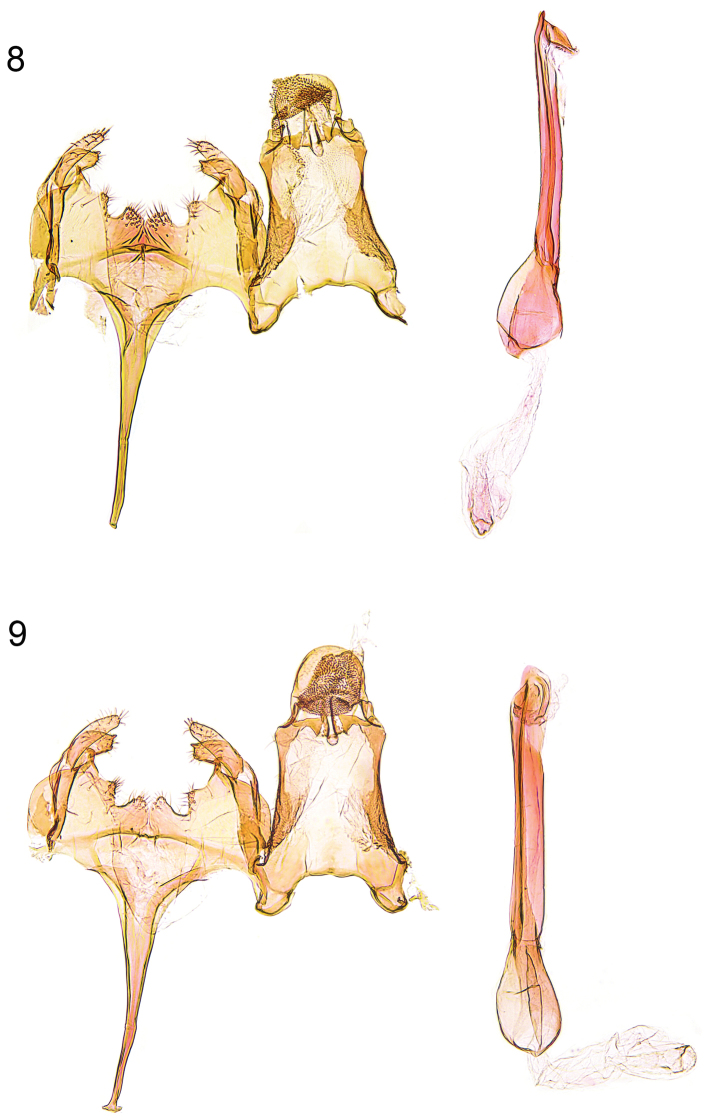
Male genitalia. **8** *Caryocolum amaurella* (Hering), Finland, slide GU 14/1373 P.Huemer; **9** *Caryocolum amaurella*, Finland, slide GU 14/1374 P.Huemer.

**Male genitalia** (Figs 8–9, 12–13). Uncus subovate; tegumen stout; transtilla membranous; valva short, moderately stout, apex rounded; sacculus short, with angular ventral and convex dorsal margin, apically pointed; posterior margin of vinculum with deep medial emargination and slight medial incision, two pairs of short processes developed; saccus long, comparatively broad at base, distal part gradually tapered; phallus long and slender, weakly curved, with some minute cornuti apically.

**Female genitalia** (Figs 16–17). Segment VIII without processes, subgenital plate sub-triangular, with numerous narrow folds, separated from sclerotized lateral plates by membranous zone; apophysis anterior slightly longer than segment VIII; antrum moderately short, about one-third to one-quarter length of apophysis anterior, broadly funnel-shaped; posterior part of ductus bursae with pair of lateral sclerites, extending to anterior third of apophysis anterior, and with two tiny sclerites anteriorly; signum with crescent-shaped base, short and stout, strongly bent hook.

**Figures 10–13.**
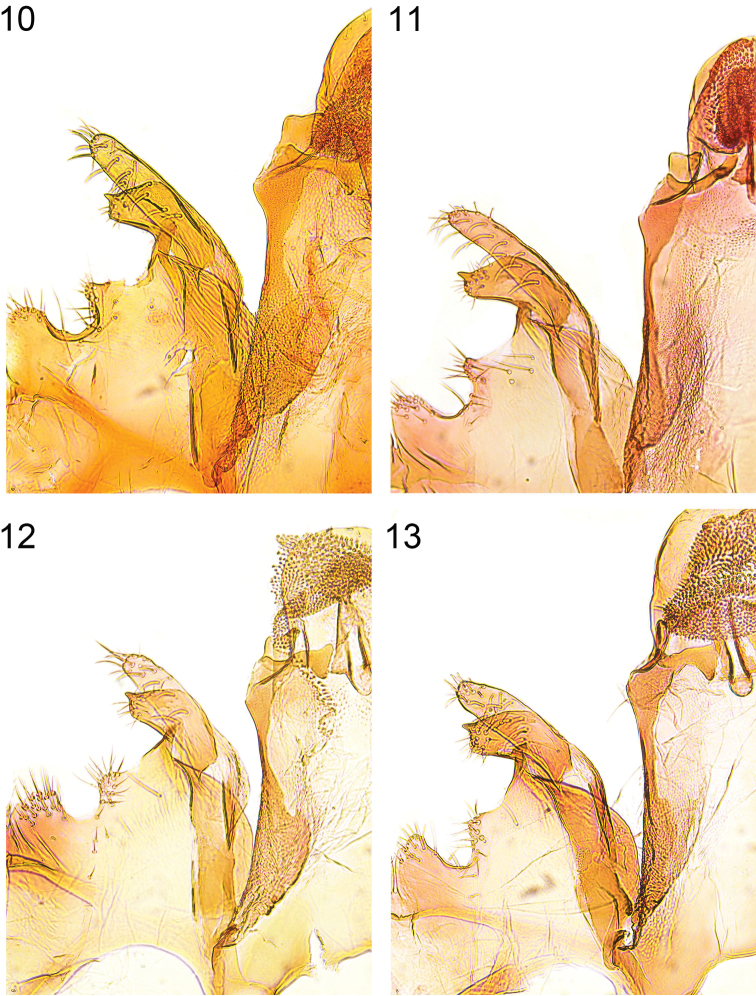
Details of male genitalia (vinculum-valva-complex). **10** *Caryocolum crypticum* sp. n., paratype, Italy, slide GU 86/041 P.Huemer **11** *Caryocolum crypticum* sp. n., paratype, Italy, slide GEL 1215 P.Huemer **12** *Caryocolum amaurella*, Finland, slide GU 14/1373 P.Huemer **13** *Caryocolum amaurella*, Finland, slide GU 14/1374 P.Huemer.

**Figures 14–15.**
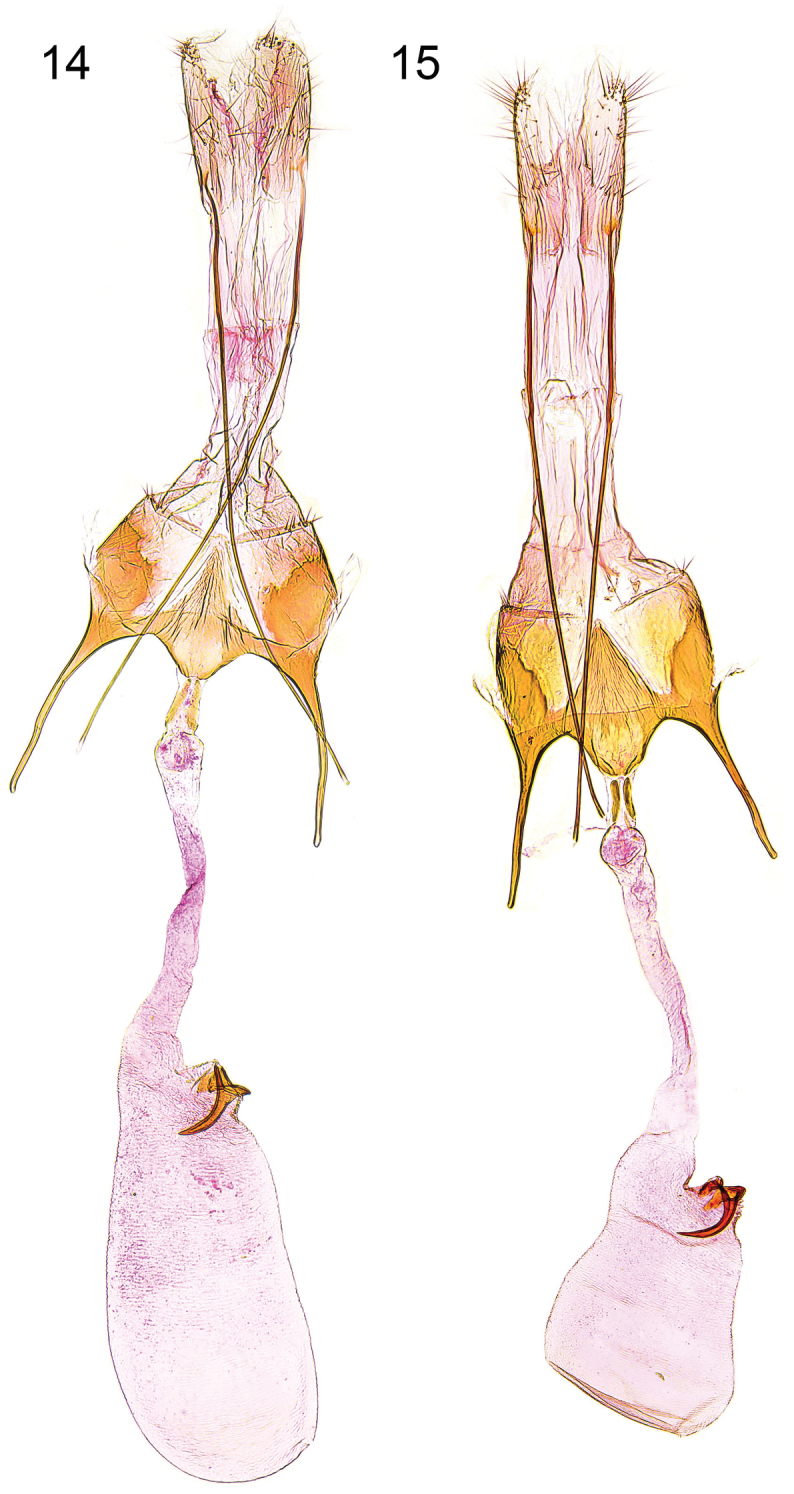
Female genitalia. **14** *Caryocolum crypticum* sp. n., holotype, slide GEL 1234 P.Huemer **15** *Caryocolum crypticum* sp. n., paratype, Italy, slide GEL 1232 P.Huemer.

**Figures 16–17.**
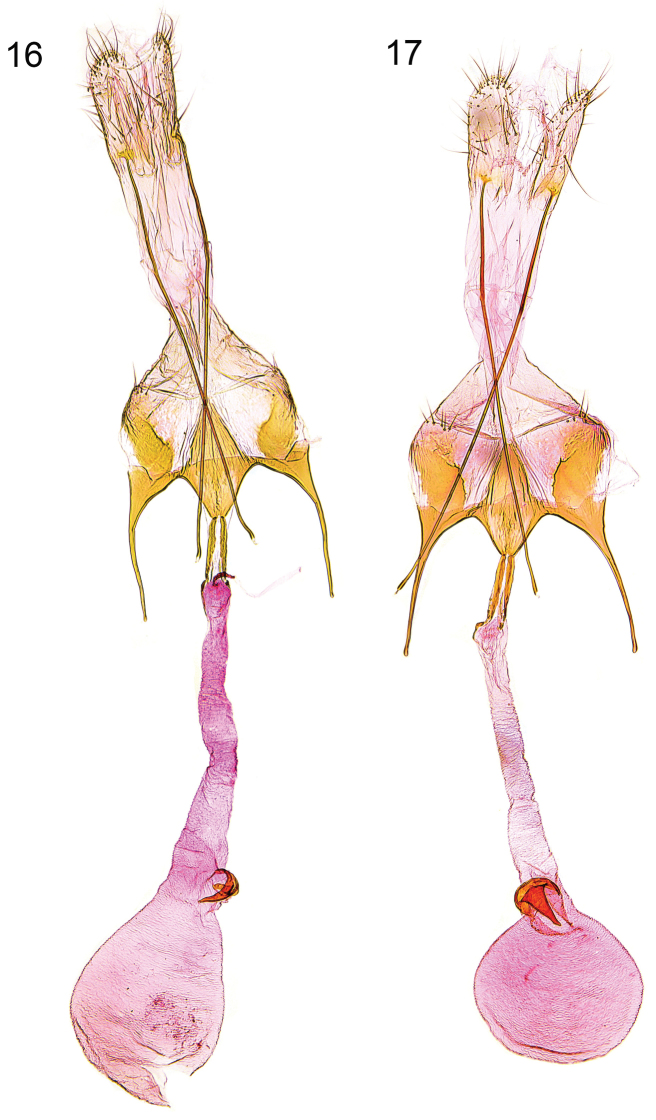
Female genitalia. **16** *Caryocolum amaurella*, Finland, slide GU 14/1372 P.Huemer **17** *Caryocolum amaurella*, Finland, slide GU 14/1371 P.Huemer.

**Molecular data.** The intraspecific divergence of the barcode region is high with mean intraspecific divergence of 3.01% and maximum intraspecific divergence of 4.62% (n=9). The distance to the nearest neighbour *Caryocolum mucronatella* is 5.21%, the divergence to the morphologically closest *Caryocolum crypticum* is 6.82%. The extraordinary high intraspecific divergence with 4 haplotypes is partially related to geographical pattern. However, we also found two haplotypes within one population in Finland and morphology does not support cryptic diversity.

##### Distribution.

With certainty known from scattered records from northern and Central Europe and Turkey. All the specimens from north of the Alps that we have been able to cross-check are correctly attributed to *Caryocolum amaurella*. However, recent records from Ukraine (Bidzilya and Budashkin 2009) and Russia (southern Ural Mountains) (Junnilainen et al. 2010) have to be re-examined due to a possible mix-up with *Caryocolum crypticum*. Records from Switzerland are dubious, and at least in one instance refer to the new species, whereas those from France (Nel 2003) are confirmed (see Huemer and Karsholt 2010, Fig. 154c).

**Bionomics.** The larva has been recorded feeding on *Silene viscaria* (L.) Jess (= *Lychnis viscaria* L. (Caryophyllaceae) (Huemer and Karsholt 2010), while the other stated host-plants, namely *Silene otites* (L.) Wibel (Burmann 1990) and *Silene nutans* L. (Huemer 1989a), refer to *Caryocolum crypticum*. Schütze (1926, 1931) gives a detailed account of the life-history. The larva feeds in April and May in the young terminal leaves which are – without spinning – attached to a tube where the larva is hidden. Dark frass is frequently extruded at the tip of the larval dwelling. Later it bores into the stem and the shoots often become swollen and stunted. Pupation takes place on the ground in a cocoon among debris. The adult occurs from late June to early September and it is attracted to light. *Caryocolum amaurella* is restricted to warm and sunny habitats such as dry meadows and pastures. Vertical distribution: from lowland localities to about 2200 m in the Alps.

##### Remarks.

*Lita amaurella* was described from an unspecified number of specimens of both sexes (‘♂, ♀’) from Finland (Bromarf) (Hering 1924). In order to stabilize nomenclature, a male, labelled as type, in ZMUH is here designated as lectotype (see data above). *Lita viscariae* was described from 67 specimens reared from *Silene viscaria* from Eastern Germany (near Rachlau) (Schütze 1926). No type material was traced during this and earlier studies (Huemer 1988), but the original descriptions and topotypical material leave no doubt about the identity.

Turkish specimens of *Caryocolum amaurella* examined by us differ from European specimens of this species by the thorax with rusty brown posterior part and the rusty brown tegulae with blackish brown base, similar to *Caryocolum crypticum*, and they are thus hardly separable from the latter on external characters. The genitalia of both sexes of *Caryocolum amaurella* from Turkey agree in all details with those of European *Caryocolum amaurella* and, because no contradicting genetic data is currently available, we consider them as belonging to that species.

One of the examined specimens of *Caryocolum amaurella* from Turkey was collected in the same locality (Kizildaĝ Geçidi, prov. Erzincan) as a specimen *Caryocolum iranicum* in ZMUC. The latter species, which is only known from a few specimens, differs, as stated above, in characters of the male genitalia.

## Discussion

The genus *Caryocolum* is a rare example of European Microlepidoptera which has gained significant attention from specialists during the last decades. Several monographic papers, from Klimesch (1953–54) to Huemer and Karsholt (2010), are a sound base for a stable taxonomy and a pre-requisite to test congruence of classical morphologically-driven species delineation with that of molecular data. DNA barcoding has evolved as a widely accepted method for preliminary species delimitation (Monaghan et al. 2009, Hendrich et al. 2010, Kekkonen and Hebert 2014) and therefore the animal DNA barcode region seemed an appropriate genetic marker to be used for this purpose. Indeed, barcoding resulted in an excellent support for all of the 44 studied species with a distinct barcode gap to the nearest neighbour ranging from about 3% to nearly 7% interspecific divergence.

Intraspecific variation shows a different pattern. The majority of species has a low (<2%) maximum intraspecific divergence and thus seems taxonomically well defined. However, a remarkable number of species (8 species, nearly one quarter of all, 9 species with only one sample not considered) is characterized by maximum divergence exceeding 3% (Fig. 1). Such deep intraspecific splits often suggest the possibility of cryptic diversity (for examples in Lepidoptera, see Dinca et al. 2011, Hausmann et al. 2009, Huemer and Hebert 2011, Huemer et al. 2012, Huemer et al 2013, Kaila and Mutanen 2012, Landry and Hebert 2013, Mutanen et al. 2012a, b, 2013, Segerer et al. 2011, Wilson et al. 2010). A morphological cross-check in one of these taxa, *Caryocolum amaurella*, proved the existence of a hitherto overlooked species with validity independently supported by morphology, biological data, and the DNA barcode. The potential of DNA barcoding for screening of cryptic diversity is obvious in this case, where morphological characters, particularly the normally well-separated male genitalia, are weak and thus have been neglected so far. Although deep intraspecific splits may alternatively refer to mitochondrial introgression, historical polymorphism or *Wolbachia* infection (Hurst and Jiggins 2005, Funk and Omland 2003), there is a considerable possibility of further cryptic diversity in the genus. In *Caryocolum schleichi* it seems most appropriate that the three sequenced subspecies should be considered as different species since host-plants and genitalia morphology differ as well (see i.e. Huemer and Karsholt 2010). The subspecies of *Caryocolum schleichi* are geographically isolated making their delimitation both rather artificial and very sensitive to the species concept applied (Mutanen et al. 2012c). An integrative revision of this group is in preparation by the authors. In contrast, the expected low divergence in subspecies is reflected by a very low divergence in *Caryocolum marmorea* and its subspecies *Caryocolum marmorea mediocorsa*. Diagnostic morphological characters seem present in further taxa from first examined samples, namely *Caryocolum fibigerium* and *Caryocolum peregrinella* with a maximum intrapecific divergence of 6.27% and 5.69% related to three deep phylogeographic splits in both species. Similar deep splits are observed in *Caryocolum alsinella* and in *Caryocolum cauligenella*. For all these taxa with subtle character differences a careful re-examination of morphology has to be undertaken in the future.

## Supplementary Material

XML Treatment for Caryocolum crypticum

XML Treatment for Caryocolum amaurella

## Supplementary material

Supplementary material 1 Sample information for specimens included in this study. doi: 10.3897/zookeys.404.7234.app1 Data type: species data Explanation note: Process IDs are sequence identifiers in BOLD; Sample IDs are specimen identifiers; BINs are Barcode Identification Numbers in BOLD. Details of collecting data, images, sequences, and trace files for the barcoded specimens are available in the public BOLD dataset “DS-LECARY”, accessed at dx.doi.org/10.5883/DS-LECARY. This dataset is made available under the Open Database License (http://opendatacommons.org/licenses/odbl/1.0/). The Open Database License (ODbL) is a license agreement intended to allow users to freely share, modify, and use this Dataset while maintaining this same freedom for others, provided that the original source and author(s) are credited.Peter Huemer, Ole Karsholt, Marko Mutanen
