# Supplementary material for: DNA barcoding as a screening tool for cryptic diversity: an example from Caryocolum, with description of a new species (Lepidoptera, Gelechiidae)
Source: Zookeys. 2014 Apr 24;(404):91–111. doi: 10.3897/zookeys.404.7234 (PMC4023261; doi:10.3897/zookeys.404.7234)
Supplement: Supplementary material 1 — Sample information for specimens included in this study. [file zookeys-404-091-s001.docx]

**Supplementary material 1**

Sample information for specimens included in this study. Process IDs are sequence identifiers in BOLD; Sample IDs are specimen identifiers; BINs are Barcode Identification Numbers in BOLD. Details of collecting data, images, sequences, and trace files for the barcoded specimens are available in the public BOLD dataset “DS-LECARY”, accessed at dx.doi.org/10.5883/DS-LECARY.

| **Species** | **Process ID** | **Sample ID** | **BIN** | **GenBank** | **Institution** |
| --- | --- | --- | --- | --- | --- |
| Caryocolum albifaciella | PHLAA747-09 | TLMF Lep 00787 | BOLD:AAK2832 | HM426134 | Tiroler Landesmuseum Ferdinandeum |
| Caryocolum albifaciella | PHLAD562-11 | TLMF Lep 03737 | BOLD:AAK2832 | JN271003 | Tiroler Landesmuseum Ferdinandeum |
| Caryocolum albifaciella | PHLAD580-11 | TLMF Lep 03755 | BOLD:AAK2832 | JN271013 | Tiroler Landesmuseum Ferdinandeum |
| Caryocolum albifaciella | PHLAD563-11 | TLMF Lep 03738 | BOLD:AAK2832 | JN271004 | Tiroler Landesmuseum Ferdinandeum |
| Caryocolum alsinella | LEEUA388-11 | MM19796 | BOLD:AAV0572 | JN270934 | University of Oulu |
| Caryocolum alsinella | PHLAE073-11 | TLMF Lep 04293 | BOLD:AAV7764 | JN271064 | Tiroler Landesmuseum Ferdinandeum |
| Caryocolum amaurella | LEFIF522-10 | MM12154 | BOLD:AAE9474 | HM875207 | University of Oulu |
| Caryocolum amaurella | LEFIF979-10 | MM13564 | BOLD:AAE9474 | HM875660 | University of Oulu |
| Caryocolum amaurella | PHLAE399-11 | TLMF Lep 04524 | BOLD:ACE5885 | KJ427049 | Tiroler Landesmuseum Ferdinandeum |
| Caryocolum amaurella | LEFIF976-10 | MM13561 | BOLD:AAE9473 | HM875657 | University of Oulu |
| Caryocolum amaurella | PHLAE398-11 | TLMF Lep 04523 | BOLD:ACE5885 | KJ427046 | Tiroler Landesmuseum Ferdinandeum |
| Caryocolum amaurella | PHLAF108-11 | TLMF Lep 05278 | BOLD:ABA2355 | KJ427129 | Tiroler Landesmuseum Ferdinandeum |
| Caryocolum amaurella | LEFIF977-10 | MM13562 | BOLD:AAE9474 | HM875658 | University of Oulu |
| Caryocolum amaurella | LEFIF978-10 | MM13563 | BOLD:AAE9474 | HM875659 | University of Oulu |
| Caryocolum amaurella | PHLAF107-11 | TLMF Lep 05277 | BOLD:ABA2355 | KJ427133 | Tiroler Landesmuseum Ferdinandeum |
| Caryocolum blandella | LEFIF465-10 | MM11987 | BOLD:AAI6556 | HM875150 | University of Oulu |
| Caryocolum blandella | LEFIJ1299-11 | MM21159 | BOLD:AAI6556 | KJ427043 | University of Oulu |
| Caryocolum blandella | LEFIB755-10 | MM02608 | BOLD:AAI6556 | HM871632 | University of Oulu |
| Caryocolum blandella | LEFIC179-10 | MM03533 | BOLD:AAI6556 | HM872024 | University of Oulu |
| Caryocolum blandelloides | PHLAI021-12 | KLM Lep 00496 | BOLD:AAK2834 | KJ427031 | Landesmuseum Kaernten |
| Caryocolum blandelloides | LEFIF059-10 | MM10499 | BOLD:AAK2834 | HM874770 | University of Oulu |
| Caryocolum blandelloides | LEFIK668-10 | MM18243 | BOLD:AAK2834 | JF854237 | University of Oulu |
| Caryocolum blandelloides | LEFIK150-10 | MM17725 | BOLD:AAK2834 | KJ427078 | University of Oulu |
| Caryocolum blandulella | PHLAI019-12 | KLM Lep 00494 | BOLD:AAV7765 | KJ427087 | Landesmuseum Kaernten |
| Caryocolum blandulella | LEFIL286-10 | MM19286 | BOLD:AAV7765 | JN270954 | University of Oulu |
| Caryocolum blandulella | LEFIL287-10 | MM19287 | BOLD:AAV7765 | KJ427098 | University of Oulu |
| Caryocolum blandulella | LEEUA184-11 | MM19592 | BOLD:AAV7765 | KJ427095 | University of Oulu |
| Caryocolum blandulella | PHLAI020-12 | KLM Lep 00495 | BOLD:AAV7765 | KJ427075 | Landesmuseum Kaernten |
| Caryocolum cassella | PHLAE405-11 | TLMF Lep 04530 | BOLD:AAE9472 | KJ427045 | Tiroler Landesmuseum Ferdinandeum |
| Caryocolum cassella | PHLAI151-12 | KLM Lep 00626 | BOLD:AAE9472 | KJ427121 | Landesmuseum Kaernten |
| Caryocolum cassella | LEFIF586-10 | MM12412 | BOLD:AAE9472 | HM875270 | University of Oulu |
| Caryocolum cassella | LEFIE633-10 | MM09575 | BOLD:AAE9472 | HM874356 | University of Oulu |
| Caryocolum cassella | LEFIE046-10 | MM08149 | BOLD:AAE9472 | HM873794 | University of Oulu |
| Caryocolum cassella | LEFIE707-10 | MM09699 | BOLD:AAE9472 | HM874428 | University of Oulu |
| Caryocolum cauligenella | LEFIJ776-10 | MM17401 | BOLD:AAP7513 | JF853817 | University of Oulu |
| Caryocolum cauligenella | LEATC404-13 | TLMF Lep 12386 | BOLD:AAP7513 | KJ427056 | Tiroler Landesmuseum Ferdinandeum |
| Caryocolum cauligenella | LEFIJ778-10 | MM17403 | BOLD:AAP7513 | JF853819 | University of Oulu |
| Caryocolum cauligenella | LEATC403-13 | TLMF Lep 12385 | BOLD:AAP7513 | KJ427035 | Tiroler Landesmuseum Ferdinandeum |
| Caryocolum cauligenella | PHLSA084-11 | TLMF Lep 03259 | BOLD:AAU3080 | JN271117 | Tiroler Landesmuseum Ferdinandeum |
| Caryocolum cauligenella | LEFIJ777-10 | MM17402 | BOLD:AAP7513 | JF853818 | University of Oulu |
| Caryocolum cauligenella | LEATD655-13 | TLMF Lep 13302 | BOLD:AAP7513 | KJ427119 | Tiroler Landesmuseum Ferdinandeum |
| Caryocolum confluens | PHLAG580-12 | TLMF Lep 07259 | BOLD:ABW9623 | KJ427060 | Tiroler Landesmuseum Ferdinandeum |
| Caryocolum crypticum | LEATC331-13 | TLMF Lep 12313 | BOLD:ACJ5624 | KJ427040 | Tiroler Landesmuseum Ferdinandeum |
| Caryocolum crypticum | LEATE470-13 | TLMF Lep 11882 | BOLD:ACJ5624 | KJ427122 | Tiroler Landesmuseum Ferdinandeum |
| Caryocolum crypticum | LEATE471-13 | TLMF Lep 11883 | BOLD:ACJ5624 | KJ427124 | Tiroler Landesmuseum Ferdinandeum |
| Caryocolum dauphini | PHLAI446-13 | TLMF Lep 08941 | BOLD:ACE2159 | KJ427050 | Tiroler Landesmuseum Ferdinandeum |
| Caryocolum dauphini | PHLAI447-13 | TLMF Lep 08942 | BOLD:ACE2159 | KJ427081 | Tiroler Landesmuseum Ferdinandeum |
| Caryocolum delphinatella | PHLAB677-10 | TLMF Lep 01477 | BOLD:AAK2827 | HQ968691 | Tiroler Landesmuseum Ferdinandeum |
| Caryocolum delphinatella | LEEUA478-11 | MM20537 | BOLD:AAK2827 | KJ427114 | University of Oulu |
| Caryocolum delphinatella | PHLAB676-10 | TLMF Lep 01476 | BOLD:AAK2827 | HQ968690 | Tiroler Landesmuseum Ferdinandeum |
| Caryocolum delphinatella | PHLAA057-09 | TLMF Lep 00097 | BOLD:AAK2827 | HM432291 | Tiroler Landesmuseum Ferdinandeum |
| Caryocolum fibigerium | PHLAI014-12 | KLM Lep 00489 | BOLD:ACC2659 | KJ427116 | Landesmuseum Kaernten |
| Caryocolum fibigerium | PHLAI013-12 | KLM Lep 00488 | BOLD:ACC2659 | KJ427062 | Landesmuseum Kaernten |
| Caryocolum fibigerium | PHLSA083-11 | TLMF Lep 03258 | BOLD:AAU3076 | JN271116 | Tiroler Landesmuseum Ferdinandeum |
| Caryocolum fibigerium | PHLAG510-12 | TLMF Lep 06904 | BOLD:AAU3076 | KJ427071 | Tiroler Landesmuseum Ferdinandeum |
| Caryocolum fibigerium | PHLSA082-11 | TLMF Lep 03257 | BOLD:AAU3076 | JN271115 | Tiroler Landesmuseum Ferdinandeum |
| Caryocolum fibigerium | PHLAE533-11 | TLMF Lep 05038 | BOLD:AAO2674 | KJ427112 | Tiroler Landesmuseum Ferdinandeum |
| Caryocolum fibigerium | PHLAB801-10 | TLMF Lep 01601 | BOLD:AAO2674 | HQ968809 | Tiroler Landesmuseum Ferdinandeum |
| Caryocolum fibigerium | PHLAB800-10 | TLMF Lep 01600 | BOLD:AAO2674 | HQ968808 | Tiroler Landesmuseum Ferdinandeum |
| Caryocolum fibigerium | PHLAI015-12 | KLM Lep 00490 | BOLD:ACC2659 | KJ427062 | Landesmuseum Kaernten |
| Caryocolum fibigerium | PHLAI403-13 | TLMF Lep 08898 | BOLD:AAU3076 | KJ427120 | Tiroler Landesmuseum Ferdinandeum |
| Caryocolum fibigerium | PHLAI404-13 | TLMF Lep 08899 | BOLD:AAU3076 | KJ427068 | Tiroler Landesmuseum Ferdinandeum |
| Caryocolum fischerella | LEFIF972-10 | MM13556 | BOLD:AAI6565 | HM875653 | University of Oulu |
| Caryocolum fischerella | LEFIJ1243-11 | MM21103 | BOLD:AAI6565 | KJ427027 | University of Oulu |
| Caryocolum fischerella | LEFIF971-10 | MM13555 | BOLD:AAI6565 | HM875652 | University of Oulu |
| Caryocolum fischerella | LEFIJ1517-12 | MM22785 | BOLD:AAI6565 | KJ427101 | University of Oulu |
| Caryocolum fraternella | PHLAG506-12 | TLMF Lep 06900 | BOLD:AAI6571 | KJ427079 | Tiroler Landesmuseum Ferdinandeum |
| Caryocolum fraternella | LEFIA843-10 | MM09719 | BOLD:AAI6571 | HM386983 | University of Oulu |
| Caryocolum fraternella | LEFIC851-10 | MM05066 | BOLD:AAI6571 | HM872669 | University of Oulu |
| Caryocolum fraternella | LEFIF970-10 | MM13552 | BOLD:AAI6571 | HM875651 | University of Oulu |
| Caryocolum fraternella | PHLAG505-12 | TLMF Lep 06899 | BOLD:AAI6571 | KJ427090 | Tiroler Landesmuseum Ferdinandeum |
| Caryocolum fraternella | LEFIF480-10 | MM12032 | BOLD:AAI6571 | HM875165 | University of Oulu |
| Caryocolum gallagenellum | PHLAE428-11 | TLMF Lep 04553 | BOLD:ABV4975 | KJ427036 | Tiroler Landesmuseum Ferdinandeum |
| Caryocolum huebneri | PHLAH147-12 | TLMF Lep 06647 | BOLD:ABW6646 | KJ427110 | Research Collection of Peter Buchner |
| Caryocolum interalbicella | PHLAA054-09 | TLMF Lep 00094 | BOLD:AAI6572 | GU689183 | Tiroler Landesmuseum Ferdinandeum |
| Caryocolum interalbicella | PHLAA055-09 | TLMF Lep 00095 | BOLD:AAI6572 | GU689184 | Tiroler Landesmuseum Ferdinandeum |
| Caryocolum interalbicella | LEATC328-13 | TLMF Lep 12310 | BOLD:AAI6572 | KJ427074 | Naturmuseum Suedtirol |
| Caryocolum interalbicella | LEATE379-13 | TLMF Lep 11791 | BOLD:AAI6572 | KJ427042 | Tiroler Landesmuseum Ferdinandeum |
| Caryocolum interalbicella | PHLAD577-11 | TLMF Lep 03752 | BOLD:AAI6572 | JN271011 | Tiroler Landesmuseum Ferdinandeum |
| Caryocolum jaspidella | PHLAG578-12 | TLMF Lep 07257 | BOLD:ABW6542 | KJ427039 | Tiroler Landesmuseum Ferdinandeum |
| Caryocolum jaspidella | PHLAG579-12 | TLMF Lep 07258 | BOLD:ABW6542 | KJ427118 | Tiroler Landesmuseum Ferdinandeum |
| Caryocolum junctella | LEATC507-13 | TLMF Lep 12489 | BOLD:AAQ1185 | KJ427125 | Tiroler Landesmuseum Ferdinandeum |
| Caryocolum junctella | LEFIL705-10 | MM05724 | BOLD:AAQ1185 | JF854695 | University of Oulu |
| Caryocolum junctella | PHLAI156-12 | KLM Lep 00631 | BOLD:AAQ1185 | KJ427094 | Landesmuseum Kaernten |
| Caryocolum klosi | PHLAG512-12 | TLMF Lep 06906 | BOLD:ABW0326 | KJ427076 | Tiroler Landesmuseum Ferdinandeum |
| Caryocolum klosi | GELWP190-11 | KLM Lep 00190 | BOLD:AAY7823 | KJ427091 | Landesmuseum Kaernten |
| Caryocolum kroesmanniella | LEFIF466-10 | MM11991 | BOLD:AAI6573 | HM875151 | University of Oulu |
| Caryocolum kroesmanniella | LEFIE482-10 | MM09258 | BOLD:AAI6573 | HM874206 | University of Oulu |
| Caryocolum kroesmanniella | PHLAG507-12 | TLMF Lep 06901 | BOLD:AAI6573 | KJ427065 | Tiroler Landesmuseum Ferdinandeum |
| Caryocolum kroesmanniella | LEFIF521-10 | MM12153 | BOLD:AAI6573 | HM875206 | University of Oulu |
| Caryocolum laceratella | PHLAB900-10 | TLMF Lep 01700 | BOLD:AAO4665 | HQ968904 | Tiroler Landesmuseum Ferdinandeum |
| Caryocolum leucomelanella | PHLAB830-10 | TLMF Lep 01630 | BOLD:AAM3503 | HQ968834 | Tiroler Landesmuseum Ferdinandeum |
| Caryocolum leucomelanella | COLFF1041-13 | MM23250 | BOLD:ACI3418 | KJ427088 | University of Helsinki |
| Caryocolum leucomelanella | LEATE421-13 | TLMF Lep 11833 | BOLD:AAM3503 | KJ427051 | Tiroler Landesmuseum Ferdinandeum |
| Caryocolum leucomelanella | PHLAB829-10 | TLMF Lep 01629 | BOLD:AAM3503 | HQ968833 | Tiroler Landesmuseum Ferdinandeum |
| Caryocolum leucomelanella | PHLAF100-11 | TLMF Lep 05270 | BOLD:AAM3503 | KJ427073 | Tiroler Landesmuseum Ferdinandeum |
| Caryocolum leucomelanella | PHLAD578-11 | TLMF Lep 03753 | BOLD:AAM3503 | JN271012 | Tiroler Landesmuseum Ferdinandeum |
| Caryocolum leucomelanella | PHLAF105-11 | TLMF Lep 05275 | BOLD:AAM3503 | KJ427059 | Tiroler Landesmuseum Ferdinandeum |
| Caryocolum leucothoracellum | PHLAB802-10 | TLMF Lep 01602 | BOLD:AAO3805 | HQ968810 | Tiroler Landesmuseum Ferdinandeum |
| Caryocolum leucothoracellum | PHLAD013-11 | TLMF Lep 02998 | BOLD:AAO3805 | JN270991 | Tiroler Landesmuseum Ferdinandeum |
| Caryocolum leucothoracellum | LEATE455-13 | TLMF Lep 11867 | BOLD:AAO3805 | KJ427030 | Tiroler Landesmuseum Ferdinandeum |
| Caryocolum leucothoracellum | PHLAB803-10 | TLMF Lep 01603 | BOLD:AAO3805 | HQ968811 | Tiroler Landesmuseum Ferdinandeum |
| Caryocolum leucothoracellum | LEATE422-13 | TLMF Lep 11834 | BOLD:AAO3805 | KJ427104 | Tiroler Landesmuseum Ferdinandeum |
| Caryocolum marmorea | LEEUA182-11 | MM19590 | BOLD:AAK2828 | KJ427041 | University of Oulu |
| Caryocolum marmorea | LEATD659-13 | TLMF Lep 13306 | BOLD:ACJ5938 | KJ427111 | Tiroler Landesmuseum Ferdinandeum |
| Caryocolum marmorea | LEATC292-13 | TLMF Lep 12274 | BOLD:ACJ5938 | KJ427113 | Naturmuseum Suedtirol |
| Caryocolum marmorea | PHLAB831-10 | TLMF Lep 01631 | BOLD:ACJ5938 | HQ968835 | Tiroler Landesmuseum Ferdinandeum |
| Caryocolum marmorea mediocorsa | PHLAI202-13 | TLMF Lep 08956 | BOLD:AAK2828 | KJ427126 | Research Collection of Thierry Varenne |
| Caryocolum marmorea mediocorsa | PHLAI203-13 | TLMF Lep 08957 | BOLD:AAK2828 | KJ427100 | Research Collection of Thierry Varenne |
| Caryocolum mazeli | PHLAG331-12 | TLMF Lep 06725 | BOLD:ABY3315 | KJ427093 | Tiroler Landesmuseum Ferdinandeum |
| Caryocolum mucronatella | LEATC402-13 | TLMF Lep 12384 | BOLD:AAW4102 | KJ427097 | Tiroler Landesmuseum Ferdinandeum |
| Caryocolum mucronatella | LEATC400-13 | TLMF Lep 12382 | BOLD:AAW4102 | KJ427084 | Tiroler Landesmuseum Ferdinandeum |
| Caryocolum mucronatella | PHLAD620-11 | TLMF Lep 03795 | BOLD:AAW4102 | KJ427099 | Tiroler Landesmuseum Ferdinandeum |
| Caryocolum mucronatella | PHLAH548-12 | TLMF Lep 08367 | BOLD:AAW4102 | KJ427083 | Tiroler Landesmuseum Ferdinandeum |
| Caryocolum oculatella | PHLAE427-11 | TLMF Lep 04552 | BOLD:ABV4976 | KJ427054 | Tiroler Landesmuseum Ferdinandeum |
| Caryocolum peregrinella | LEATA364-13 | TLMF Lep 09781 | BOLD:ACK3061 | KJ427055 | Research Collection of R. J. Heckford |
| Caryocolum peregrinella | PHLAA608-09 | TLMF Lep 00648 | BOLD:AAC6816 | HM381457 | Tiroler Landesmuseum Ferdinandeum |
| Caryocolum peregrinella | PHLAB612-10 | TLMF Lep 01412 | BOLD:AAC6816 | HQ968639 | Tiroler Landesmuseum Ferdinandeum |
| Caryocolum peregrinella | PHLAB278-10 | TLMF Lep 01078 | BOLD:AAC6817 | HM381634 | Tiroler Landesmuseum Ferdinandeum |
| Caryocolum peregrinella | PHLAB206-10 | TLMF Lep 01006 | BOLD:AAC6816 | HM381570 | Tiroler Landesmuseum Ferdinandeum |
| Caryocolum peregrinella | PHLAB623-10 | TLMF Lep 01423 | BOLD:AAC6818 | HQ968647 | Tiroler Landesmuseum Ferdinandeum |
| Caryocolum peregrinella | PHLAF186-11 | TLMF Lep 05356 | BOLD:AAC6818 | KJ427117 | Tiroler Landesmuseum Ferdinandeum |
| Caryocolum peregrinella | LEATE240-13 | TLMF Lep 11652 | BOLD:AAC6817 | KJ427070 | Naturmuseum Suedtirol |
| Caryocolum peregrinella | LEATE239-13 | TLMF Lep 11651 | BOLD:AAC6817 | KJ427128 | Tiroler Landesmuseum Ferdinandeum |
| Caryocolum peregrinella | PHLAF187-11 | TLMF Lep 05357 | BOLD:AAC6818 | KJ427096 | Tiroler Landesmuseum Ferdinandeum |
| Caryocolum peregrinella | PHLAB621-10 | TLMF Lep 01421 | BOLD:AAC6817 | HQ968645 | Tiroler Landesmuseum Ferdinandeum |
| Caryocolum peregrinella | PHLAI017-12 | KLM Lep 00492 | BOLD:AAC6818 | KJ427107 | Landesmuseum Kaernten |
| Caryocolum peregrinella | PHLAA512-09 | TLMF Lep 00552 | BOLD:AAC6817 | HM381394 | Tiroler Landesmuseum Ferdinandeum |
| Caryocolum peregrinella | PHLAB276-10 | TLMF Lep 01076 | BOLD:AAC6818 | HQ968453 | Tiroler Landesmuseum Ferdinandeum |
| Caryocolum peregrinella | PHLAI018-12 | KLM Lep 00493 | BOLD:AAC6818 | KJ427086 | Landesmuseum Kaernten |
| Caryocolum peregrinella | LEATA365-13 | TLMF Lep 09782 | BOLD:ACK3061 | KJ427066 | Research Collection of R. J. Heckford |
| Caryocolum peregrinella | PHLAB622-10 | TLMF Lep 01422 | BOLD:AAC6817 | HQ968646 | Tiroler Landesmuseum Ferdinandeum |
| Caryocolum peregrinella | PHLAB624-10 | TLMF Lep 01424 | BOLD:AAC6818 | HQ968648 | Tiroler Landesmuseum Ferdinandeum |
| Caryocolum peregrinella | PHLAA053-09 | TLMF Lep 00093 | BOLD:AAC6816 | HM432290 | Tiroler Landesmuseum Ferdinandeum |
| Caryocolum petrophila | LEATD657-13 | TLMF Lep 13304 | BOLD:AAO3809 | KJ427069 | Naturmuseum Suedtirol |
| Caryocolum petrophila | LEFIK002-10 | MM17577 | BOLD:AAO3809 | JX034639 | University of Oulu |
| Caryocolum petrophila | PHLAB895-10 | TLMF Lep 01695 | BOLD:AAO3809 | HQ968899 | Tiroler Landesmuseum Ferdinandeum |
| Caryocolum petrophila | LEFIK397-10 | MM17972 | BOLD:AAO3809 | JF854029 | University of Oulu |
| Caryocolum petrophila | PHLAC678-10 | TLMF Lep 02713 | BOLD:AAO3809 | JF860239 | Tiroler Landesmuseum Ferdinandeum |
| Caryocolum petrophila | PHLAB896-10 | TLMF Lep 01696 | BOLD:AAO3809 | HQ968900 | Tiroler Landesmuseum Ferdinandeum |
| Caryocolum petrophila | LEFIK398-10 | MM17973 | BOLD:AAO3809 | JF854030 | University of Oulu |
| Caryocolum petrophila | LEFIJ1014-11 | MM09288 | BOLD:AAO3809 | KJ427089 | University of Oulu |
| Caryocolum petrophila | PHLAF099-11 | TLMF Lep 05269 | BOLD:AAO3809 | JX034690 | Tiroler Landesmuseum Ferdinandeum |
| Caryocolum petrophila | LEATD658-13 | TLMF Lep 13305 | BOLD:AAO3809 | KJ427103 | Tiroler Landesmuseum Ferdinandeum |
| Caryocolum petryi | PHLAB897-10 | TLMF Lep 01697 | BOLD:AAM0549 | HQ968901 | Tiroler Landesmuseum Ferdinandeum |
| Caryocolum petryi | LEFIG784-10 | MM15648 | BOLD:AAM0549 | HM876436 | University of Oulu |
| Caryocolum petryi | LEFIJ775-10 | MM17400 | BOLD:AAM0549 | JF853816 | University of Oulu |
| Caryocolum petryi | PHLAB1109-10 | TLMF Lep 01909 | BOLD:AAM0549 | HQ968325 | Tiroler Landesmuseum Ferdinandeum |
| Caryocolum petryi | PHLAB1111-10 | TLMF Lep 01911 | BOLD:AAM0549 | HQ968326 | Tiroler Landesmuseum Ferdinandeum |
| Caryocolum petryi | PHLAC679-10 | TLMF Lep 02714 | BOLD:AAM0549 | JF860240 | Tiroler Landesmuseum Ferdinandeum |
| Caryocolum petryi | LEFIJ774-10 | MM17399 | BOLD:AAM0549 | JF853815 | University of Oulu |
| Caryocolum petryi | PHLAB1110-10 | TLMF Lep 01910 | BOLD:AAM0549 | JF859623 | Tiroler Landesmuseum Ferdinandeum |
| Caryocolum proxima | PHLAD020-11 | TLMF Lep 03005 | BOLD:AAH4687 | JN270994 | Tiroler Landesmuseum Ferdinandeum |
| Caryocolum proxima | LEATD656-13 | TLMF Lep 13303 | BOLD:AAH4687 | KJ427108 | Tiroler Landesmuseum Ferdinandeum |
| Caryocolum proxima | LEATA569-13 | TLMF Lep 10176 | BOLD:AAH4687 | KJ427127 | Naturmuseum Suedtirol |
| Caryocolum pullatella | LEFIB936-10 | MM03085 | BOLD:AAC1599 | HM871813 | University of Oulu |
| Caryocolum pullatella | PHLAC841-10 | TLMF Lep 02876 | BOLD:AAC1598 | JF860338 | Tiroler Landesmuseum Ferdinandeum |
| Caryocolum pullatella | PHLAC838-10 | TLMF Lep 02873 | BOLD:AAC1598 | KJ427028 | Tiroler Landesmuseum Ferdinandeum |
| Caryocolum pullatella | PHLAF106-11 | TLMF Lep 05276 | BOLD:AAC1598 | JX034697 | Tiroler Landesmuseum Ferdinandeum |
| Caryocolum pullatella | LEFIB286-10 | MM00778 | BOLD:AAC1599 | HM871187 | University of Oulu |
| Caryocolum pullatella | LEFIE429-10 | MM09053 | BOLD:AAC1599 | HM874153 | University of Oulu |
| Caryocolum pullatella | PHLAF103-11 | TLMF Lep 05273 | BOLD:AAC1598 | JX034655 | Tiroler Landesmuseum Ferdinandeum |
| Caryocolum pullatella | LEFIG527-10 | MM14646 | BOLD:AAC1599 | HM876200 | University of Oulu |
| Caryocolum pullatella | PHLAF102-11 | TLMF Lep 05272 | BOLD:AAC1598 | JX034621 | Tiroler Landesmuseum Ferdinandeum |
| Caryocolum repentis | PHLAD576-11 | TLMF Lep 03751 | BOLD:AAV7766 | JN271010 | Tiroler Landesmuseum Ferdinandeum |
| Caryocolum repentis | LEATA496-13 | TLMF Lep 10103 | BOLD:AAV7766 | KJ427115 | Tiroler Landesmuseum Ferdinandeum |
| Caryocolum repentis | PHLAD575-11 | TLMF Lep 03750 | BOLD:AAV7766 | JN271009 | Tiroler Landesmuseum Ferdinandeum |
| Caryocolum repentis | PHLAF466-11 | TLMF Lep 05636 | BOLD:AAV7766 | KJ427034 | Tiroler Landesmuseum Ferdinandeum |
| Caryocolum saginella | PHLAA069-09 | TLMF Lep 00109 | BOLD:AAI6574 | HM425738 | Tiroler Landesmuseum Ferdinandeum |
| Caryocolum saginella | PHLAA585-09 | TLMF Lep 00625 | BOLD:AAI6574 | HM381455 | Tiroler Landesmuseum Ferdinandeum |
| Caryocolum schleichi arenariella | LEFIG783-10 | MM15647 | BOLD:AAE9479 | HM876435 | University of Oulu |
| Caryocolum schleichi arenariella | LEFIG782-10 | MM15646 | BOLD:AAE9479 | HM876434 | University of Oulu |
| Caryocolum schleichi arenariella | LEFIL552-10 | MM18850 | BOLD:AAE9479 | JF854655 | University of Oulu |
| Caryocolum schleichi arenariella | PHLAF104-11 | TLMF Lep 05274 | BOLD:AAE9479 | KJ427044 | Tiroler Landesmuseum Ferdinandeum |
| Caryocolum schleichi arenariella | LEFIJ779-10 | MM17404 | BOLD:AAE9479 | JF853820 | University of Oulu |
| Caryocolum schleichi dianthella | PHLSA085-11 | TLMF Lep 03260 | BOLD:AAU1854 | KJ427064 | Tiroler Landesmuseum Ferdinandeum |
| Caryocolum schleichi improvisella | PHLAA067-09 | TLMF Lep 00107 | BOLD:AAE9478 | HM432294 | Tiroler Landesmuseum Ferdinandeum |
| Caryocolum schleichi improvisella | PHLAD574-11 | TLMF Lep 03749 | BOLD:AAE9478 | JN271008 | Tiroler Landesmuseum Ferdinandeum |
| Caryocolum schleichi improvisella | PHLAD573-11 | TLMF Lep 03748 | BOLD:AAE9478 | JN271007 | Tiroler Landesmuseum Ferdinandeum |
| Caryocolum schleichi improvisella | PHLAD572-11 | TLMF Lep 03747 | BOLD:AAE9478 | JN271006 | Tiroler Landesmuseum Ferdinandeum |
| Caryocolum schleichi improvisella | PHLAA493-09 | TLMF Lep 00533 | BOLD:AAE9478 | HM381377 | Tiroler Landesmuseum Ferdinandeum |
| Caryocolum siculum | PHLAE429-11 | TLMF Lep 04554 | BOLD:ABV4977 | KJ427109 | Tiroler Landesmuseum Ferdinandeum |
| Caryocolum srnkai | PHLAF489-11 | TLMF Lep 05659 | BOLD:ABU7385 | KJ427047 | Research Collection of Peter Sonderegger |
| Caryocolum tischeriella | LEFIC281-10 | MM03744 | BOLD:AAI6566 | HM872125 | University of Oulu |
| Caryocolum tischeriella | PHLAE266-11 | TLMF Lep 04486 | BOLD:AAI6566 | JN271085 | Tiroler Landesmuseum Ferdinandeum |
| Caryocolum tischeriella | LEATE424-13 | TLMF Lep 11836 | BOLD:ACK2775 | KJ427058 | Tiroler Landesmuseum Ferdinandeum |
| Caryocolum tischeriella | LEFIC280-10 | MM03743 | BOLD:AAI6566 | HM872124 | University of Oulu |
| Caryocolum tischeriella | PHLAE265-11 | TLMF Lep 04485 | BOLD:AAI6566 | JN271084 | Tiroler Landesmuseum Ferdinandeum |
| Caryocolum tischeriella | PHLAI157-12 | KLM Lep 00632 | BOLD:ACE2964 | KJ427061 | Landesmuseum Kaernten |
| Caryocolum tischeriella | LEATC401-13 | TLMF Lep 12383 | BOLD:ACE2964 | KJ427077 | Tiroler Landesmuseum Ferdinandeum |
| Caryocolum tischeriella | LEATE425-13 | TLMF Lep 11837 | BOLD:ACE2964 | KJ427052 | Tiroler Landesmuseum Ferdinandeum |
| Caryocolum tischeriella | LEATC405-13 | TLMF Lep 12387 | BOLD:ACE2964 | KJ427048 | Tiroler Landesmuseum Ferdinandeum |
| Caryocolum tischeriella | PHLAF101-11 | TLMF Lep 05271 | BOLD:AAI6566 | KJ427134 | Tiroler Landesmuseum Ferdinandeum |
| Caryocolum trauniella | PHLAB898-10 | TLMF Lep 01698 | BOLD:AAO3810 | HQ968902 | Tiroler Landesmuseum Ferdinandeum |
| Caryocolum trauniella | PHLAB899-10 | TLMF Lep 01699 | BOLD:AAO3810 | HQ968903 | Tiroler Landesmuseum Ferdinandeum |
| Caryocolum tricolorella | LEFIF968-10 | MM13550 | BOLD:AAF1506 | HM875649 | University of Oulu |
| Caryocolum tricolorella | LEFIF467-10 | MM11992 | BOLD:AAF1506 | HM875152 | University of Oulu |
| Caryocolum tricolorella | LEFIF969-10 | MM13551 | BOLD:AAF1506 | HM875650 | University of Oulu |
| Caryocolum vicinella | LEFIF973-10 | MM13557 | BOLD:AAL6797 | HM875654 | University of Oulu |
| Caryocolum vicinella | LEATC350-13 | TLMF Lep 12332 | BOLD:ACC3292 | KJ427106 | Tiroler Landesmuseum Ferdinandeum |
| Caryocolum vicinella | LEFIF975-10 | MM13559 | BOLD:AAL6797 | HM875656 | University of Oulu |
| Caryocolum vicinella | PHLAI155-12 | KLM Lep 00630 | BOLD:ACC3292 | KJ427123 | Landesmuseum Kaernten |
| Caryocolum vicinella | LEFIF974-10 | MM13558 | BOLD:AAL6797 | HM875655 | University of Oulu |
| Caryocolum vicinella | LEATE469-13 | TLMF Lep 11881 | BOLD:ACC3292 | KJ427132 | Tiroler Landesmuseum Ferdinandeum |
| Caryocolum vicinella | PHLAE531-11 | TLMF Lep 05036 | BOLD:ABA3050 | KJ427029 | Tiroler Landesmuseum Ferdinandeum |
| Caryocolum vicinella | PHLAI153-12 | KLM Lep 00628 | BOLD:ACC3292 | KJ427130 | Landesmuseum Kaernten |
| Caryocolum viscariella | PHLAE409-11 | TLMF Lep 04534 | BOLD:AAI6568 | KJ427067 | Tiroler Landesmuseum Ferdinandeum |
| Caryocolum viscariella | PHLAE410-11 | TLMF Lep 04535 | BOLD:AAI6568 | KJ427131 | Tiroler Landesmuseum Ferdinandeum |
| Caryocolum viscariella | LEFIL695-10 | MM18993 | BOLD:AAI6568 | KJ427037 | University of Oulu |
| Caryocolum viscariella | PHLAE408-11 | TLMF Lep 04533 | BOLD:AAI6568 | KJ427072 | Tiroler Landesmuseum Ferdinandeum |
| Caryocolum viscariella | PHLAC263-10 | TLMF Lep 02298 | BOLD:AAI6568 | JF859869 | Tiroler Landesmuseum Ferdinandeum |
| Caryocolum viscariella | PHLAH554-12 | TLMF Lep 08373 | BOLD:AAI6568 | KJ427082 | Tiroler Landesmuseum Ferdinandeum |
| Caryocolum viscariella | PHLAD036-11 | TLMF Lep 03021 | BOLD:AAI6568 | JN270997 | Tiroler Landesmuseum Ferdinandeum |
| Caryocolum viscariella | LEFIF469-10 | MM11997 | BOLD:AAI6568 | HM875154 | University of Oulu |
| Caryocolum viscariella | PHLAD029-11 | TLMF Lep 03014 | BOLD:AAI6568 | JN270996 | Tiroler Landesmuseum Ferdinandeum |
| Caryocolum viscariella | LEFIF470-10 | MM11998 | BOLD:AAI6568 | HM875155 | University of Oulu |
| Caryocolum viscariella | LEFIB285-10 | MM00777 | BOLD:AAI6568 | KJ427085 | University of Oulu |
